# Mutations in an avian IgY-Fc fragment reveal the locations of monocyte Fc receptor binding sites

**DOI:** 10.1016/j.dci.2009.08.012

**Published:** 2010-02

**Authors:** Alexander I. Taylor, Brian J. Sutton, Rosaleen A. Calvert

**Affiliations:** Randall Division of Cell and Molecular Biophysics, King's College London, New Hunt's House, Guy's Campus, London SE1 1UL, United Kingdom

**Keywords:** Cα/ɛ/γ/υ, heavy chain constant domain of IgA/IgE/IgG/IgY, CHIR-AB1, chicken leukocyte immunoglobulin-like receptor AB1, FcαR, the leukocyte receptor for IgA (CD89), FcγRIII, a low affinity receptor for IgG (CD16), FcɛRI, the high-affinity receptor for IgE, Fcυ2–4, chicken IgY-Fc fragment containing heavy chain constant domains 2, 3 and 4, MQ-NCSU, a chicken monocyte cell line, sfpCHIR-AB1, soluble fusion protein of the extracellular region of CHIR-AB1 and human IgG-Fc, SPR, surface plasmon resonance (Biacore), Antibodies, Birds, Evolution, Fc receptors, Immunity, Immunoglobulins

## Abstract

The avian IgY antibody isotype shares a common ancestor with both mammalian IgG and IgE and so provides a means to study the evolution of their structural and functional specialisations. Although both IgG and IgE bind to their leukocyte Fc receptors with 1:1 stoichiometry, IgY binds to CHIR-AB1, a receptor expressed in avian monocytes, with 2:1 stoichiometry. The mutagenesis data reported here explain the structural basis for this difference, mapping the CHIR-AB1 binding site to the Cυ3/Cυ4 interface and not the N-terminal region of Cυ3 where, at equivalent locations, the IgG and IgE leukocyte Fc receptor binding sites lie. This finding, together with the phylogenetic relationship of the antibodies and their receptors, indicates that a substantial shift in the nature of Fc receptor binding occurred during the evolution of mammalian IgG and IgE.

## 1 Introduction

Interactions between the Fc region of immunoglobulins (Ig) and membrane-bound Fc receptors on cells of the innate immune system are key, isotype-specific events in the activation and regulation of the vertebrate immune system. A wide variety of immune responses may be tailored to a particular antigenic challenge through control over which Ig isotype is secreted and which Fc receptors are expressed on each cell type [1]. The evolution of novel or improved functions in vertebrate adaptive immune systems is therefore closely tied to the appearance and co-evolution of new Ig isotypes and Fc receptors.

Birds and reptiles possess an Ig isotype called IgY, which is functionally analogous to IgG of mammals: both are present in the serum at high levels (∼10 mg/mL) and provide defence against microbial infection. A duplication of the gene encoding an IgY-like heavy chain occurred between 160 and 310 mya, during the evolution of mammals, and allowed the divergence of both IgG and IgE [2,3], the latter of which is involved in anti-parasitic responses and allergic hypersensitivity. As this did not occur in the bird/reptile lineage, the ancestral isotype has been conserved; comparative studies with IgY therefore offer a means to deduce the evolutionary changes that have allowed IgG and IgE to adapt to their different roles in modern species [4]. Although IgY is functionally similar to IgG, its structure appears to have conserved features of both IgG and IgE [5,6].

IgG and IgE have several leukocyte Fc receptors, most of which are closely related (e.g. FcγRI-IV, FcɛRI); in humans, the classical Fc receptor gene cluster (FcR) is found on chromosome 1, together with a variety of related Fc receptor-like (FCRL) sequences that are thought to be immunoregulatory receptors, but do not bind IgG or IgE [7]. In birds, this cluster is represented by a single gene [8,9] encoding a receptor-like molecule that does not bind IgY. Instead, the leukocyte IgY-Fc receptors identified to date belong to a large group of similar genes, chicken immunoglobulin-like receptors (CHIR), which are homologous to the leukocyte receptor cluster (LRC) on human chromosome 19, but only distantly related to FcR/FCRL [10]. In order to reconcile the phylogeny of IgY, IgG and IgE with that of their Fc receptors, we previously postulated that a major evolutionary event must have occurred: the migration of Fc receptor function from one gene family to another, perhaps driven by selection pressure to evade microbial peptides that compete with Fc receptor binding [6]. An ‘arms race’ of this sort has been shown to drive diversification of the leukocyte Fc receptor binding site in IgA, another Ig isotype more distantly related to IgY that is involved in mucosal immunity [11]. Although FcR/FCRL genes are present in basal amphibians, phylogenetic analysis of the FcR/FCRL gene cluster indicates that the non-Fc-binding FCRLs are the more ancient members of the cluster [8,9,12], which supports a relatively recent acquisition of Fc receptor function for this cluster (i.e. mammalian FcRs appear to have evolved from non-Fc-binding ancestors).

In order to further investigate what may therefore be a primitive Fc receptor interaction, we have mapped the Fc receptor binding site on an avian (chicken, *Gallus gallus*) IgY-Fc, using mutagenesis to identify key amino acids involved in binding to an avian monocyte cell line, MQ-NCSU [13], and to the soluble extracellular region of CHIR-AB1, a high-affinity activating IgY receptor that is expressed on several avian leukocytes including monocytes [14].

## 2 Materials and methods

### 2.1 Generation of mutant IgY-Fc fragments and soluble CHIR-AB1

A cDNA cassette encoding the Fcυ2–4 fragment of chicken (*G gallus*) IgY (residues 230–568), with an N-terminal signal peptide to allow secretion, was cloned as described previously [6] and ligated into pCEP4 (Invitrogen). Site-directed mutagenesis was performed using Quikchange (Stratagene). Plasmids were transiently transfected into HEK293E cells using TransIT (Mirus Bio) and culture medium supplemented with 0.2 mg/mL Hygromycin B (Invitrogen) to effect selection. Secreted IgY-Fc fragments were isolated from the cell culture medium using anti-IgY-Fc-agarose (Immunology Consultants Laboratory) and further purified by size exclusion chromatography using Superdex 200 (GE Life Sciences), as described previously [6]. sfpCHIR-AB1, a soluble fusion protein consisting of the extracellular domain of CHIR-AB1 and the Fc region of human IgG1 [14], was prepared using the above expression system, as described previously [15].

### 2.2 Flow cytometry (FACS) analysis of mutant IgY-Fc fragments binding to monocytes

Chicken MQ-NCSU monocytes [13] were assayed for IgY-Fc binding as described previously [5]. Briefly, cells were incubated with purified IgY-Fc mutants (10 nM, saturating for wild-type at 4 °C) or buffer (phosphate-buffered saline with 1% BSA) alone for 1 h, then stained with mouse monoclonal anti-IgY-Fc (Cυ2-specific) antibodies (Sigma) and anti-mouse-IgG-FITC (Dako) sequentially, with prior washes. Cells were analysed using a FACSCalibur instrument (BD Biosciences).

### 2.3 SPR analysis of mutant IgY-Fc fragments binding to CHIR-AB1

IgY-Fc binding to sfpCHIR was assayed as described previously [15]. Briefly, 90RU of purified sfpCHIR-AB1 were immobilised on a CM5 sensor surface and binding of purified IgY-Fc (Fcυ2–4) mutants (25–400 nM) was measured using a Biacore 3000 instrument. Association rate constants (*k*_*on*_), dissociation rate constants (*k*_*off*_) and equilibrium association constants (*K*_A_) were obtained by fitting with a 1:1 binding model.

## 3 Results and discussion

**Fig. 1:**
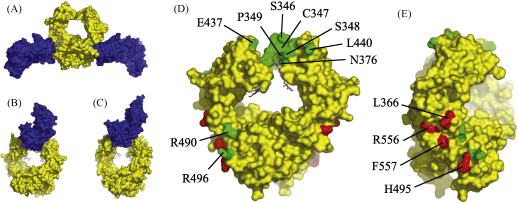
Space-filling diagrams showing the Fc fragments (yellow) of human IgA (A), IgE (B) and IgG (C) in complex with their leukocyte Fc receptors (blue), FcαRI, FcɛRI and FcγRIII, respectively, and two orthogonal views (D and E) of chicken IgY-Fc (domains Cυ3 and Cυ4). Residues 346–348 were modelled onto the crystal structure of chicken Fcυ3–4 for display purposes, although these residues were found to be disordered [5]. Cυ2 domains are not shown, although they were included in the IgY-Fc used in this study. Residues whose mutation had no effect are coloured green and labelled in (D); residues whose mutation inhibited binding to soluble CHIR-AB1 and/or MQ-NCSU monocytes are coloured red and labelled in (E).

Previous research has suggested that interactions between avian IgY and its Fc receptors may be more similar to that of human IgA and its leukocyte Fc receptor, FcαR, than the equivalent interactions between IgG and IgE and their leukocyte Fc receptors; both FcαR and CHIR-AB1 are encoded in the LRC, their Fc-binding domains are structurally homologous (rmsd = 1.9 Å) and soluble monomeric versions of each have been found to bind to their ligands with 2:1 stoichiometry [15,16]. By contrast, the leukocyte receptors for both IgG and IgE bind with 1:1 stoichiometry. The structural basis of this difference has been revealed by the location of the Fc receptor binding site in each isotype [17]; the binding site for FcαRI in IgA-Fc lies at the interface of the Cα2 and Cα3 domains (Fig. 1A), whereas both IgE-Fc and IgG-Fc engage their leukocyte receptors in similar locations close to the N-termini of the homologous Cɛ3 and Cγ2 domains, respectively (Fig. 1B and C).

In order to investigate whether the equivalent of either region in IgY-Fc is the location of an Fc receptor binding site, candidate receptor binding residues were selected for mutation by examining the crystal structures of chicken IgY-Fc (PDB ID 2W59) and CHIR-AB1 (PDB ID 2VSD), superposed on the complexes of IgA-Fc, IgG-Fc or IgE-Fc bound to FcαRI (PDB ID 1OW0), FcγRIII (PDB 1T83) and FcɛRI (PDB ID 1O0V), respectively. To limit the number of candidate residues, those conserved between IgY and IgG and/or IgE were ignored as neither IgG nor IgE bind to avian monocytes [18]. All residues were mutated individually, except 346–349 inclusive and 437/440, which were mutated together, for reasons of practicality. The locations of the mutated residues are shown in Fig. 1D and E.

**Fig. 2:**
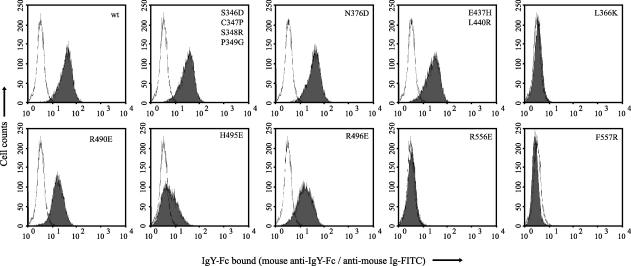
FACS histograms showing binding of chicken IgY-Fc mutants to chicken monocytes. MQ-NCSU cells were incubated with IgY-Fc (Fcυ2–4) fragments containing the mutations shown (filled peaks), or buffer alone (unfilled peaks), then stained for bound IgY-Fc (see Section 2).

**Fig. 3:**
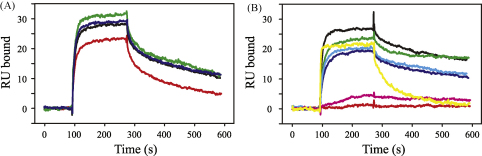
SPR sensorgrams showing binding of chicken IgY-Fc mutants to soluble chicken CHIR-AB1. IgY-Fc (Fcυ2–4) fragments were tested on a CM5 chip coated with sfpCHIR-AB1, a soluble fusion protein consisting of the extracellular domain of CHIR-AB1 and the Fc region of human IgG1 [14]. Data obtained for 400 nM of each IgY-Fc mutant are shown as follows: (A) wild-type (black), S346D/C347P/S348R/P349G (red), N376D (green), E437H/L440R (blue); (B) wild-type (black), L366K (red), R490E (green), H495E (blue), R496E (cyan), R556E (magenta), F557R (yellow).

The results show that mutations made close to the Cυ3 N-terminus had little or no effect on binding to either monocytes (Fig. 2) or soluble CHIR-AB1 (Table 1 and Fig. 3), whereas mutation of L366, H495, R556 or F557, all of which lie close to the interface between Cυ3 and Cυ4 in the crystal structure of IgY-Fc, was found to markedly disturb binding to monocytes. Binding to soluble CHIR-AB1 was also affected by these mutations (note the severely reduced binding for L366K and R556E, and markedly enhanced dissociation rate for F557R, reflected in the grossly different shapes of the traces in Fig. 3B), with the exception of H495E, which appeared to have wild-type binding kinetics. As the diminished binding of IgY-Fc H495E to monocytes cannot be accounted for by abrogated binding to CHIR-AB1, yet incubation with excess soluble CHIR-AB1 can block IgY-Fc binding to monocytes [15], this suggests the presence of other high-affinity Fc receptors with binding sites that overlap that of CHIR-AB1 on avian monocytes. Viertlboeck et al. have recently described several novel IgY-Fc receptors that are highly similar to CHIR-AB1, although their expression patterns are currently unknown [19]. Our results show that the location of the IgY-Fc binding site for avian Fc receptors on monocytes, including CHIR-AB1, lies in a similar region to the binding site for FcαRI in IgA-Fc and not in the equivalent region to the FcR binding sites in IgG or IgE.

Although it is possible that any mutation may have an unforeseen allosteric effect as, for example, occurs when residues in or close to the EF or AB helices in the Cɛ3 domains of IgE-Fc (which lie at the Cɛ3/Cɛ4 interface and do not make direct contact with receptor) are non-conservatively mutated [20,21], the blocking mutations identified in the present study covered a broad surface area and included conservative substitutions which, in the case of the IgE-Fc studies, was sufficient to eliminate ‘false-positives’, i.e. mutations that affected receptor binding despite lying outside the binding site [22].

The majority of the receptor contacts in IgA-Fc are found in the Cα3 FG loop, CC′ loop and the Cα2 AB helix [23]; the homologous structures in IgY-Fc also appear to be involved in binding to IgY-Fc receptors. We note that these regions are well conserved in other bird and reptile IgY sequences in the literature (Supplementary Fig. 1), which suggests that similarly interacting CHIR-AB1 orthologues may be present in these species. Despite such widespread conservation between chicken and duck IgY sequences, duck IgY fails to bind to chicken CHIR-AB1 [14]. However, this is likely due to a single amino acid difference between the duck and chicken Cυ3 AB helix, rather than a completely different mode of interaction; the duck sequence contains an arginine at a position adjacent to that of leucine 366 in the chicken, one of the residues which, when substituted for a charged residue (lysine), was found to prevent binding to both monocytes and soluble CHIR-AB1 entirely (Figs. 2 and 3). It is therefore likely that both chicken and duck IgY interact with their respective receptors in a broadly similar manner: the positive residue in the duck sequence being accommodated by a complementary site in the duck receptor, whilst other pairings remain identical. However, more substantial differences in the mode of interaction cannot be ruled out. In order to investigate the likelihood that the interaction between IgY and its leukocyte receptor(s) predates the divergence of the bird/reptile class, all available amphibian IgY sequences were also compared with chicken IgY, but were found to be poorly conserved in the receptor binding regions identified above (Supplementary Fig. 1). This divergence may be due to the evolutionary distance between birds and amphibians or reflect the different (predominantly mucosal) functions served by IgY and its receptors in amphibians [24].

The findings reported here are consistent with the 2:1 stoichiometry for monomeric CHIR-AB1 reported previously [15] and explain the lack of involvement of intra-heavy chain disulphide bonds [18], the Cυ2 domains [6] and N-linked glycosylation [5] in binding to avian monocytes. By contrast, similar studies of mammalian IgG and IgE have shown that the equivalent structural features are crucial to the mechanics of their receptor interactions: in IgE, intra-heavy chain disulphide bonds [25] and the Cɛ2 domains [26] are required to establish the exceptionally slow off-rate that leads to sensitisation of mast cells and basophils; in IgG, complex-type N-linked oligosaccharides that lie between the Cγ2 domains affect the structure of IgG-Fc and the affinity of FcγR interactions [27,28], which in turn modulates the effector response [29]. The involvement of these structural features in the functions of IgE and IgG is dependent on the location of their Fc receptor binding sites, which are similar in both mammalian isotypes (Fig. 1B and C), yet, as the results presented here show, substantially different in IgY.

Our results provide evidence for a major shift in the mode of Fc receptor binding during the evolution of vertebrate immunoglobulins and show how various structural features of antibody Fc regions (e.g. inter-chain disulphide bonds and glycosylation) may have been co-opted to provide novel functions during the evolution of IgG and IgE in mammals. From a strict Darwinian perspective, the abrupt acquisition of a novel receptor and binding site seems highly unorthodox; *natura non facit saltum*. However, our understanding of the evolution of protein–protein interaction networks, although still in its infancy, has revealed that enzymes can evolve novel or improved substrate specificity by capitalising on weak promiscuous interactions [30], which typically arise from neutral genetic drift. Should this principle apply to the proteins of the immune system, it would present a hitherto unappreciated evolutionary mechanism by which proteins may rapidly be co-opted for novel functions, and interaction networks ‘rewired’ to evade interfering proteins of microbial origin. In the case of mammalian IgE and IgG, such rewiring, i.e. migration of receptor binding activity to a site in the N-terminal region of Cγ2/Cɛ3, as well as interaction with a member of a different receptor family, appears to have been a key step that occurred prior to their divergence from an IgY-like ancestor and subsequently allowed existing structural features to be co-opted to novel roles as the antibodies diverged and their current specialisations evolved.

## Figures and Tables

**Table 1:** SPR measurements of recombinant IgY-Fc mutants binding to soluble CHIR-AB1.

IgY-Fc mutations	*k*_*on*_ (M^−1^ s^−1^)	*k*_*off*_ (s^−1^)	*K*_A_ (M^−1^)
wt	3.0 × 10^5^	2.1 × 10^−3^	1.4 × 10^8^
S346/C347P/S348R/P349G	4.2 × 10^5^	4.4 × 10^−3^	9.5 × 10^7^
N376D	4.1 × 10^5^	2.1 × 10^−3^	2.0 × 10^8^
E437H/L440R	3.8 × 10^5^	2.1 × 10^−3^	1.8 × 10^8^

wt^a^	2.6 × 10^5^	1.2 × 10^−3^	2.1 × 10^8^
L366K	ND^b^	ND^b^	ND^b^
R490E	2.3 × 10^5^	5.1 × 10^−4^	4.5 × 10^8^
H495E	1.7 × 10^5^	1.5 × 10^−3^	1.1 × 10^8^
R496E	2.2 × 10^5^	1.3 × 10^−3^	1.8 × 10^8^
R556E	1.1 × 10^4^	1.1 × 10^−3^	9.3 × 10^6^
F557R	9.2 × 10^5^	0.013	7.3 × 10^7^

a Wild-type Fcυ2–4 was re-measured immediately before the indicated mutants were run.

b Immeasurable as maximum binding was 1RU.
