# Supplementary material for: Mutations in an avian IgY-Fc fragment reveal the locations of monocyte Fc receptor binding sites
Source: Dev Comp Immunol. 2010 Feb;34(2):97–101. doi: 10.1016/j.dci.2009.08.012 (PMC2795851; doi:10.1016/j.dci.2009.08.012)
Supplement: Supplementary file 1 [file mmc1.pdf]

```
                                                                     Cυ3 ⟶
Chicken         SQS-PAVRSGS-TYSLSSRVNVSGTDWREGKSYSCRVRHPATNTVVEDHVKGCP-DGAQS 346
Duck            TMTRPQREAGSKTYMATSQTNVSREDWKAGKAFTCRVKHPATGGTAQGHARFCPGSGAQS
Anole           YTEPPRQDAVGYTFSTTSSANISQADWLEGTIYTCKVTHAGSQTTMRARAKKCEDDS-TS
Gecko           HTENPRKDATGDTFSTTSTANVTQIDWKEGKTYTCQVIHQG--HKVEKHATKCRGDSGNC
Axolotl         QTSPPQKDGQG-TFSTTSQINVTKSDWASGDKYTCKVEHPATSSRAEDTIHNCADSQ---
Newt            YTSPSIKGREG-TFSTTSQVNISKSDWILGERYTCKVDHPATNTSLHDSIRNCPESP---
Frog            ASVPTPSKTEDGTYSSSSQLRILKGMWNKGTQYSCIVTHTSSNTTTIANISQCTEQC---
                    .     . *:  :*  .:    *  *  ::* * * .           *  .
                                 AB helix
Chicken         CSP-IQLYAIPPSPGELYISLDAKLRCLVVNLPSDSSLSVTWTREKSGNLRPDPMVLQEH 405
Duck            CSP-IQIFVVPPSPGSLYIRQDAKVHCLVVNLPSDASLSISWTREKSGALRPDPMVLTEH
Anole           QCDGIYVYLKSPTPNDLYLNRDPKVSCVVGNLESEEGLKVSWSRDKKASLDPKPMSVSEE
Gecko           TANGIKVSILPPTPADLYMNGEPRLICVVTGLESNVGMKVRWSKEKPGTLNPEPLEPAQD
Axolotl         TPYQPKVFLIAPKARDLYIANQPVVICKITKMENSDSLSVTWKRREGPEEAAVISEQYID
Newt            SGVQLKVSMIPPKAKDLYVTKQPSIVCQVTKMESADGLSITWKQEDGSPLVGLPHETEVL
Frog            -HDNLQVYPLTPTFHDLYFSRNAKVTCLVSSMKTIENFDISWEREKAGNLEFVTEDPVLH
                      :   .*.  .**.  :. : * :  : .  .:.: * : .
                                                              Cυ4 ⟶
Chicken         FNGTYSASSAVPVSTQDWLSGERFTCTVQHEELPLPLSKSVYRNT-GPTTPPLIYPFAPR 464
Duck            FNGTFTASSSLAISTQDWLAGERFTCTVQHEDLPVPLGKSIAKHA-GKVTAPYIFTFPPH
Anole           LNGTYTVVSALPILTREWDSEETFTCTVEYPGVPTPIVKKITKLK-GKVTTPSVYLFRPH
Gecko           SDGTFTLKSPLIISKQDWLAGEIYTCTLEHSSGIAPYSKTIAKKT-GKRTQAQVYLFRPH
Axolotl         SDGTFTAMSYLNITKNEWERGDEFTCKVKHFDLPFPLSRSVSKPT-GRSFAPTMYVFAPH
Newt            PDGTLGADSTLTITTDAWKQGSAFSCKVEHPDLSSPVTRTIRKPK-EREIGPSMYLFPPH
Frog            DNGTYSVASILSVCAEDWESGDKFSCTVRSQDLPSPVKKTIFKQNEGTPKAPDVYLLPPS
                 :**    * : :    *   . ::*.:.      *  :.: :        . :: : *
                                                 CC′ loop
Chicken         PEELSLS---RVTLSCLVRGFRPRDIEIRWLRDHRAVPATEFVTTAVLPEERTANGAGGD 521
Duck            AEELSLA---EVTLTCLVRGFQPEHVEVQWLRNHNSVPAAEFVTTPPLKE------PNGD
Anole           REELIFQSHSQLTLTCLVKNLNPKDVSVQWLKDNNALTEDNHITTPVLKD--------SI
Gecko           NEELKSR-DPNVSITCLVQGFNPEDISIKWLENHNAVAGNNHVTTQVQMD--------SD
Axolotl         EMELANY--DFVSLTCLVKSFSPDDIYIQWKQGKSVIPSDKYVSMEPRQEAG-----TAG
Newt            QHELDQH--ELVSLTCLVKGFSPPDIYVQWKHRNTVLPDNSSRNTALVLESG-----TNG
Frog            AQELIQQ--EMVTLTCFVTGFNPKEIFIQWMQGGVSISEDKFINTVPMKS--------DG
                  **       ::::*:* .: * .: ::* .    :.  .  .     .
                                                 FG loop
Chicken         GDTFFVYSKMSVETAKWNGGTVFACMAVHEALPMRFSQRTLQKQAGK-----------   568
Duck            G-TFFLYSKMTVPKASWQGGVSYACMVVHEGLPMRFTQRPLQKTPGK-----------
Anole           EDSYFVYSKLLISRADWDRGSSYTCMVIHEGLAMKFTQRTVEKSQGKK----------
Gecko           QDSFFVYSKLTVPKANWNDGHSFTCHVVHEGFSMKYTQRTIEKTQGKK----------
Axolotl         LGTYFSYSMLTIQKSDWDKRETFTCVAAHSAVPKNLMTRRIQKPLGK-----------
Newt            PDTYFMYSLLTISKSNWENRDTYSCIAFHSALPKNQMQRSIQKARGN-----------
Frog            EQTYFIYSKLAIPAAKWNQGDVFTCVVGHEALPLYITQQSIDKSSGKSSLVNVNLVMS
                  ::* ** : :  :.*:    ::* . *....     : ::*  *:
```

Supplementary Fig. 1. Amino acid sequences of upsilon (IgY) heavy chain sequences from birds, reptiles and amphibians, aligned using ClustalW [1]. Residues in chicken IgY-Fc involved in binding to monocytes and/or CHIR-AB1, and conserved residues in other species, are highlighted. Exact matches are coloured red, similar residues are coloured yellow.

Reference

[1] Chenna R, Sugawara H, Koike T, Lopez R, Gibson TJ, Higgins DG, Thompson JD. Multiple sequence alignment with the Clustal series of programs. Nucleic Acids Res 2003; 31:3497-3500.
